# The Human Tyrosyl-DNA Phosphodiesterase 1 (hTdp1) Inhibitor NSC120686 as an Exploratory Tool to Investigate Plant *Tdp1* Genes

**DOI:** 10.3390/genes9040186

**Published:** 2018-03-28

**Authors:** Anca Macovei, Andrea Pagano, Maria Elisa Sabatini, Sofia Grandi, Alma Balestrazzi

**Affiliations:** Department of Biology and Biotechnology ‘L. Spallanzani’, University of Pavia, via Ferrata 9, 27100 Pavia, Italy; andrea.pagano01@universitadipavia.it (A.P.); maeli_89@hotmail.com (M.E.S.); sofia.grandi01@universitadipavia.it (S.G.); alma.balestrazzi@unipv.it (A.B.)

**Keywords:** Tyrosyl-DNA phosphodiesterase, *Medicago truncatula*, NSC120686, DNA damage

## Abstract

The hTdp1 (human tyrosyl-DNA phosphodiesterase 1) inhibitor NSC120686 has been used, along with topoisomerase inhibitors, as a pharmacophoric model to restrain the Tdp1 activity as part of a synergistic treatment for cancer. While this compound has an end-point application in medical research, in plants, its application has not been considered so far. The originality of our study consists in the use of hTdp1 inhibitor in *Medicago truncatula* cells, which, unlike human cells, contain two *Tdp1* genes. Hence, the purpose of this study was to test the hTdp1 inhibitor NSC120686 as an exploratory tool to investigate the plant *Tdp1* genes, since their characterization is still in incipient phases. To do so, *M. truncatula* calli were exposed to increasing (75, 150, 300 μM) concentrations of NSC120686. The levels of cell mortality and DNA damage, measured via diffusion assay and comet assay, respectively, were significantly increased when the highest doses were used, indicative of a cytotoxic and genotoxic threshold. In addition, the NSC120686-treated calli and untreated *MtTdp1α*-depleted calli shared a similar response in terms of programmed cell death (PCD)/necrosis and DNA damage. Interestingly, the expression profiles of *MtTdp1α* and *MtTdp1β* genes were differently affected by the NSC120686 treatment, as *MtTdp1α* was upregulated while *MtTdp1β* was downregulated. The NSC120686 treatment affected not only the *MtTdp1* genes but also other genes with roles in alternative DNA repair pathways. Since the expression patterns of these genes were different than what was observed in the *MtTdp1α*-depleted plants, it could be hypothesized that the NSC120686 treatment exerts a different influence compared to that resulting from the lack of the *MtTdp1α* gene function.

## 1. Introduction

The anticancer chemotherapeutic agent camptothecin and its derivatives act as inhibitors of DNA topoisomerase I (TopI, EC 5.99.1.2) by trapping the enzyme on DNA and converting the transient TopI-DNA cleavage complexes into permanent cytotoxic lesions [1]. Cells remove these stabilized TopI-DNA cleavage complexes through the activity of the tyrosyl-DNA phosphodiesterase 1 enzyme (Tdp1, EC 3.1.4), which catalyzes the hydrolysis of the covalent linkage between the catalytic tyrosine residue of TopI and the 3′-end of a DNA phosphodiester bond [2]. In budding yeast, TopI-mediated DNA damage is also removed by alternative pathways involving endonuclease complexes among which Rad1-Rad10 (Radiation Insensitive 1–10) [3], while in human cells the same role is carried out by the endonuclease XPF (*Xeroderma pigmentosum* Complementation Group F)-ERCC1 (Excision Repair Cross-Complementation group 1), a critical component of TC-NER (Transcription-Coupled Nucleotide Excision Repair) [4]. Cancer cells often lack these alternative pathways, thus relying only on the Tdp1-mediated repair to face TopI poisons [5]. For this reason, the combined use of TopI and Tdp1 inhibitors is currently envisaged as a promising strategy to enhance the efficacy of chemotherapy.

The strongest inhibitors of the human enzyme (hTdp1) so far identified are classified as Tdp1 phosphotyrosine substrate mimetics since they share the same structural features of the natural phosphotyrosine substrate [6]. The NSC120686 (2-chloro-6-fluorobenzaldehyde 9H-fluoren-9-ylidenehydrazone) compound tested in the present work was identified by Weidlich and colleagues [7] as a pharmacophore able to inhibit hTdp1 activity. The biological effects of NSC120686 were tested in the human ovarian carcinoma cell line IGROV-1 and in two derived sub-lines (IGROV-1CPT/L and IGROV-1CPT/H) selected for resistance to the camptothecin-derivative gimatecan. These lines showed increased *hTdp1* gene expression, confirming the involvement of Tdp1 in the cell response to the treatment [8]. Additional information concerning the biological effects of NSC120686 was provided by Al-Keilani [9] who assessed the effectiveness of a combinational therapy including hTdp1 inhibitors and TopI poisons. The NSC120686 molecule was supplied to the malignant glioma cell line U87 in presence/absence of different topoisomerase drugs. When delivered alone, the NSC120686 treatment revealed strong dose-dependent toxicity against the U87 cells while no significant correlations were observed between the *hTdp1* gene expression level and cell resistance to the inhibitor. No reports are currently available describing the effect of NSC120686 on plant cells.

The *Tdp1* gene family from *Medicago truncatula* Gaertn. has been described for the first time by Macovei and colleagues [10], while a different work characterized a *tdp1* mutant obtained by transfer DNA (tDNA) tagging in *Arabidopsis thaliana* [11]. The *MtTdp1α* and *MtTdp1β* genes were upregulated in response to heavy metal and osmotic stresses, as well as during seed imbibition when DNA repair is required to preserve genome integrity and improve seed vigor [10,12]. Transgenic *M. truncatula* plants with post-transcriptional downregulation of the *MtTdp1α* gene were subsequently obtained [13] and subjected to RNA-sequencing (RNA-seq) which highlighted differential expression of DNA damage sensing/repair and chromatin remodeling genes. Interestingly, *M. truncatula* orthologues of mammalian and yeast genes participating in repair pathways alternative to *Tdp1* were not upregulated in the *MtTdp1α*-depleted lines [13]. Additionally, Sabatini et al. [14] demonstrated that *MtTdp1α* gene depletion resulted in an overall reduction of cytosine methylation and perturbations in DNA transposon/retrotransposon expression profiles. As for the dynamics of Tdp1 enzyme inhibition in plants, it was demonstrated that both the full-length *Arabidopsis Tdp1α* complementary DNA (cDNA) and the tyrosyl-DNA phosphodiesterase (TDP) domain alone could rescue the sensitivity to the TopI inhibitor camptothecin and to vanadate analogs (inhibitors of phosphoryl-transfer reactions) in a *tdp1/rad1* mutant strain of budding yeast [15]. When exposed to vanadate derivatives (which directly bind tyrosine, mimicking phosphates or acting as transition stage analogs [16]), the *Arabidopsis tdp1* mutant plants showed significantly higher sensitivity to these compounds compared to wild-type plants [15].

The present work is based on the premise that investigating the effects of hTdp1 inhibitors in *M. truncatula* cells, a peculiar system with two distinct *Tdp1* genes, could aid to gather novel information on their roles in this model legume, with possible implications to related species of economic importance. This work represents an original perspective for exploring the DNA damage response in plants, so far never considered. In the present work, we provide evidence on the genotoxic effects of NSC120686 in plant cells using calli derived from the model legume *M. truncatula*. The study has been expanded to the *MtTdp1α*-depleted *M. truncatula* calli (Tdp1α-2a line, [13]) in order to investigate possible similarities/differences between the response to NSC120686 treatment and the response associated with *Tdp1α* gene depletion.

## 2. Materials and Methods

### 2.1. Plant Material and Treatments

Calli of *Medicago truncatula* Gaertn. cv. Jemalong (M9-10a genotype) were used in the present study. Calli were obtained from leaf explants excised from in vitro grown plants and transferred to CIM (Callus Induction Medium) containing MS (Murashige and Skoog) basal salts and vitamins [17], 3% (*w*/*v*) sucrose, 0.1 mg L^−1^ 2,4-dichlorophenoxyacetic acid (2,4-D) (Micropoli, Cesano Boscone, Italy), 0.2 mg L^−1^ zeatin (Micropoli), 2% (*v*/*v*) Gelrite™ (Micropoli), pH 5.7. Leaf explants were maintained at 23 °C in the dark for two weeks, then the resulting calli were transferred to fresh CIM medium and sub-cultured every four weeks. The *M. truncatula* transgenic line Tdp1α-2a, characterized by *Tdp1α* gene depletion induced by RNA interference (RNAi)-mediated post-transcriptional downregulation, was produced in a previous study [13]. Calli of the Tdp1α-2a line were obtained as described above and maintained in CIM medium supplemented with 50 mg L^−1^ kanamycin (Micropoli). The NSC120686 inhibitor of the hTdp1 enzyme [7] was kindly provided by the Drug Synthesis and Chemistry Branch, Developmental Therapeutics Program, Division of Cancer Treatment and Diagnosis, National Cancer Institute, Bethesda, MD, USA. NSC120686 dissolved in 10% dimethyl sulfoxide (DMSO) (dimethyl sulfoxide, Sigma-Aldrich, Milan, Italy) was added to the CIM medium and calli were maintained under these conditions for four days. The treatments were applied to calli at their exponential growth phase, 10 days after sub-culturing. Three different NSC120686 concentrations were tested: 75 μM (referred to as NSC 75), 150 μM (referred to as NSC 150), and 300 μM (referred to as NSC 300). Adequate controls, consisting of 0 μM NSC120686 (referred to as non-treated (NT) as well as treatment with DMSO 10% (referred to as DMSO control (CTRL)), were used in all experiments.

### 2.2. DNA Diffusion Assay

A DNA diffusion assay was performed on *M. truncatula* calli treated with the above mentioned NSC120686 concentrations to evaluate cell death events, namely programmed cell death (PCD) and necrosis. Nuclei were extracted as previously described [18,19]. Agarose precoated slides were prepared by spreading 1% agarose (1 mL) onto previously degreased slides and dried overnight at room temperature. Aliquots (300 μL) of nuclei suspension were mixed with 200 μL of low melting point (LMP) agarose (Sigma-Aldrich) in phosphate-buffered saline (PBS) maintained at 38 °C and two aliquots (120 μL) were gently placed/slide. The gel was covered with a cover glass and slides were cooled on ice for 10 min. Cover glasses were removed and slides were immersed in lysing solution (2.5 M NaCl, 100 mM EDTA, 10 mM Tris HCl, pH 7.5) for 20 min at room temperature. After lysis, slides were washed twice in neutral solution TBE (Tris-borate-EDTA) (89 mM Tris base, 89 mM Boric Acid, 2 mM EDTA, pH 8.3) for five min and rinsed in 70% ethanol (*v*/*v*) for 10 min at room temperature. Slides were air-dried and stored at room temperature overnight and subsequently stained with 20 μL 4′,6-diamidino-2-phenylindole (DAPI) 1 mg mL^−1^ stock solution, before scoring. An Olympus BX51 fluorescence microscope (Olympus Italia S.R.L., Milan, Italy) with an excitation filter of 340–380 nm and a barrier filter of 400 nm was used for imaging. Images were captured using an Olympus MagnaFire camera equipped with Olympus Cell^F software (Olympus Italia S.R.L.). One hundred nuclei per slide were analyzed. Cells undergoing PCD or necrosis were distinguished from viable cells as indicated [20]. Three different classes of nuclei were assigned as follows: class 0, representing viable (circular, intact) nuclei; class 1, with nuclei showing a PCD-type of morphology, characterized by homogeneous outline without any clear boundary due to nucleosomal-sized DNA diffusing into the agarose; and class 2, containing nuclei with a necrotic-type of morphology, characterized by a non-homogeneous halo appearance.

Two methods of data representation/calculation were used:

1. Cell death calculated as arbitrary units (a.u.) using the formula (adapted from [21]):

[Σ(N_c_ × c) × 100]/N_tot_ (1)

where N_c_ = number of nuclei of each class, c = the class number (e.g., 0, 1, 2), and N_tot_ = total number of counted nuclei;

2. Percentage of nuclear morphology, calculated using the formula (adapted from [20]):

(N_c_ × 100)/N_tot_ (2)

Data are represented using both types of representations, as the a.u. summarize the overall cell mortality, while the percentage of nuclei morphology distinguished between the presence of PCD- or necrosis-type of events. Aside from the negative controls (NT, DMSO CTRL), a positive control consisting of calli treated with heat shock (HS) at 95 °C for 20 min to induce high mortality rates, was also used. For each treatment, three replicated samples were analyzed in two independent experiments.

### 2.3. Single Cell Gel Electrophoresis

For single cell gel electrophoresis (SCGE), also known as comet assay, nuclei were extracted from *M. truncatula* treated and untreated calli as previously described [18,19]. The resulting suspension containing purified nuclei was mixed in equal volume with a solution containing 1% LMP agarose in PBS buffer maintained at 38 °C. Two drops of the resulting suspension were then pipetted onto agarose-precoated slides and solidified on ice. For alkaline SCGE, slides were first incubated for 30 min in alkaline buffer (1 mM Na_2_EDTA, 300 mM NaOH, pH 13) at 4 °C and then electrophoresed in the same buffer for 25 min at 0.72 V cm^−1^ in a cold chamber under dark conditions. After electrophoresis, slides were washed in 0.4 M Tris HCl pH 7.5 three times for 5 min, rinsed in 70% ethanol (*v*/*v*) three times for 5 min at 4 °C and dried overnight at room temperature. Slides were stained with 20 μL DAPI 1 mg mL^−1^ (Sigma-Aldrich). Aside from the negative controls (CTRL, DMSO), a positive control consisting of calli treated with HS (95 °C for 20 min) to induce high levels of DNA damage, was also used. For each slide, one hundred nucleoids were scored, visualized using an Olympus BX51 fluorescence microscope with an excitation filter of 340–380 nm and a barrier filter of 400 nm. Images were captured using an MagnaFire camera (Olympus Italia S.R.L.) equipped with Olympus Cell^F software. Nucleoids were classified as described [21], where each type of nuclei morphology belongs to a class from 0 to 4. The results were expressed in a.u. (see Formula (1)) to represent the overall trend in the accumulation of DNA damage.

### 2.4. RNA Extraction, Complementary DNA Synthesis, and Quantitative Real-Time Polymerase Chain Reaction Analysis

RNA isolation from treated and untreated *M. truncatula* calli was isolated as previously described [22]. Total RNA was quantified by agarose gel electrophoresis and spectrophotometric analysis using a WPA Biowave DNA (Biochrom, Cambridge, UK). One microgram of RNA was reverse-transcribed using the RevertAid First Strand cDNA Synthesis Kit (Thermo Fisher, Monza, Italy), while quantitative real-time polymerase chain reaction (qRT-PCR) was carried out using the Maxima SYBR Green qPCR Master Mix (Thermo Fisher) as indicated by the supplier. Ct values and qRT-PCR efficiency, obtained by the Rotor-Gene 6000 Series Software 1.7 (Corbett Robotics, Brisbane, Australia), were analyzed and statistically validated using the REST2009 Software V2.0.13 (Qiagen GmbH, Hilden, Germany). qRT-PCR was carried out in a final volume of 12 µL using a Rotor-Gene 6000 PCR apparatus (Corbett Robotics). The amplification conditions were: denaturation at 95 °C for 10 min (one cycle), followed by 45 cycles (each 95 °C for 15 s, 60 °C for 60 s). For each oligonucleotide set, a no-template control was used. *ELF1α* was used as reference gene [13,18,23,24]. The gene-specific oligonucleotide primers, designed using Primer3 (http://primer3.ut.ee/), are listed in Table 1. The PfaffI method [25] was used for the relative quantification of transcript accumulation. Relative expression values of NSC120686-treated samples were reported to the values of DMSO-treated samples. Data are presented as heatmaps generated using Multiple experiment Viewer (MeV) software v4.9.0 (http://mev.tm4.org). For each treatment, three replicated samples (technical replicates) were analyzed.

### 2.5. Bioinformatic and Statistical Analysis

The STRING computer service (http://string-db.org/) was used to determine the predicted protein-protein interaction of human and *Arabidopsis* Tdp1 proteins. The resulting accessions were further used for a BLAST (basic local alignment search tool) search analysis (https://blast.ncbi.nlm.nih.gov) in the Plant Genome Resource Phytozome v12.1.5 database (https://phytozome.jgi.doe.gov) to identify corresponding *M. truncatula* accessions. These were subsequently used to search the RNA-seq dataset previously obtained for *Tdp1α*-depleted lines [13], in order to identify which accessions were differentially expressed, with a main focus on DNA repair-related genes.

The GeneMANIA software (https://genemania.org/) was used to identify possible relationships among the genes, using the programs’ default parameters (Warde-Farley et al., 2010) [26]. The input list of genes consisted in *Arabidopsis Tdp1*, *Top1*, *Top2*, *MRE11*, *Rad50*, *ERCC1*, *MUS81*, *PARP1*, *DRT100*, and *ENDO3* gene accessions. *Arabidoposis thaliana* was used in this case as it is the only plant model represented in this tool. 

Correlation pattern searching and Principal Component Analysis (PCA) were carried out using gene expression data with online tools available at MetaboAnalyst v.4.0 (http://www.metaboanalyst.ca). The correlation pattern searching (correlation analysis performed against a given feature) used the Pearson correlation coefficients (*r*), and the *Tdp1α* gene expression was chosen as the defining feature.

For statistical analysis, data were subjected to analysis of variance (ANOVA) and the statistical significance of mean differences was determined using *t*-Student test. All analyses were performed with biological duplicates and in three technical replicates (2 × 3, *n* = 6).

## 3. Results

### 3.1. Effect of the hTdp1 Inhibitor NSC120686 on Medicago truncatula Cell Viability

**Figure 1:**
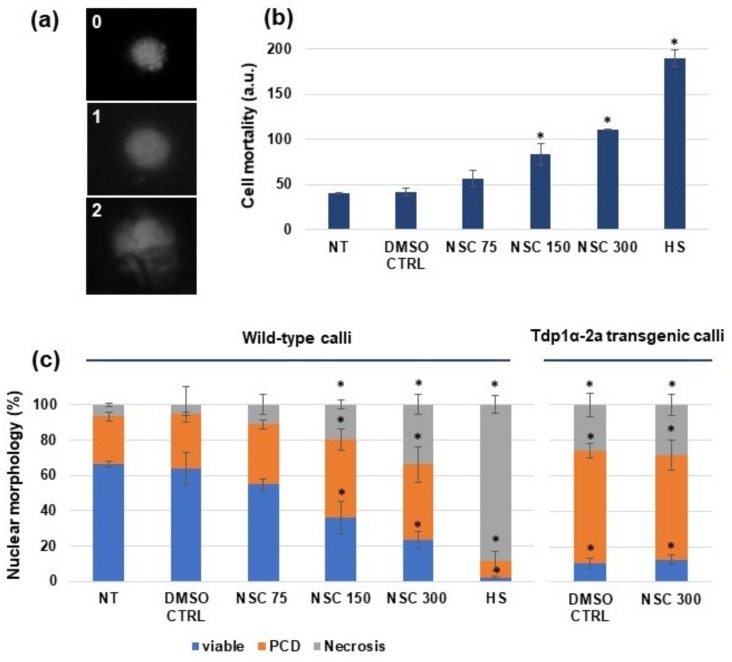
Cell viability in *Medicago truncatula* calli treated with NSC120686 as assessed by DNA diffusion assay. (**a**) Nuclear morphology of viable (0), programmed cell death (PCD) (1), and necrosis (2) events. (**b**) Cell mortality (represented in arbitrary units, a.u.) of M9-10a wild-type calli in response to increasing NSC120686 concentrations (75 μM, 150 μM, and 300 μM). (**c**) Percentage of nuclei representative for viable, PCD, and necrotic events in both wild-type and transgenic calli derived from the Tdp1α-2a line characterized by post-transcriptional downregulation of the *Tdp1α* gene. Asterisks (*) indicate significant differences (*p* < 0.05) compared to NT, as evidenced by the *t*-Student test. NT: Non-treated control; DMSO CTRL: Dimethyl sulfoxide 10% treated control; HS: Heat-shock positive control.

Wild-type *M. truncatula* calli (M9-10a genotype) and calli derived from the Tdp1α-2a line were treated with the NSC120686 compound, as described in Materials and Methods. Subsequently, a DNA diffusion assay was performed to evaluate cell viability vs. PCD and necrosis rates. Images of nuclear morphology characteristic for viable (0), PCD (1), and necrotic (2) events are presented in Figure 1a. A significant increase in cell mortality was evident when using 150 and 300 μM of NSC120686 (Figure 1b). Moreover, the 300 μM treatment was responsible for inducing 50% (100 a.u.) of cell death. As the highest-class number is 2, the maximum mortality would represent 200 a.u. (calculated based on Formula (1)) and this is reached by the positive control (HS). It is worth underlining that the estimated NSC120686 concentration able to inhibit cell growth by 50% in human malignant cell lines ranged between 10 and 50 μM [8,9].

The percentage of nuclear morphology, based on the designated classes and calculated following the Formula (2), is showed in Figure 1c. For this analysis, calli derived from the Tdp1α-2a transgenic line previously obtained through RNAi [13] were also used in order to compare the inhibitory response in the two systems. The NT and DMSO CTRL samples showed a high percentage of viable nuclei (66.1 ± 1.4 and 64.1 ± 0.4, respectively). A significant decrease in cell viability was evidenced when using 150 and 300 μM of NSC120686 (36.2 ± 8.9 and 23.3 ± 4.7, respectively), concomitant with an increase in the presence of PCD (44 ± 6.2 and 42.7 ± 10.2, respectively) and necrotic (19.8 ± 2.6 and 33.9 ± 5.5, respectively) type of morphology, while the positive control (HS) presented most of the nuclei with necrotic (76.28 ± 8.2) morphology (Figure 1c). When the diffusion assay was performed on *Tdp1α*-depleted calli, the obtained values (10.6 ± 2.6% viable cells, 63.3 ± 4.1% PCD, and 26.1 ± 6.7% necrosis) had a similar pattern of response to those registered when the non-transgenic calli were treated with 300 µM NSC120686 (Figure 1c, Tdp1α-2a transgenic calli). Based on the reported data, both the treatment with hTdp1 inhibitor and the transcriptional knockdown of the *Tdp1α* gene resulted in enhanced PCD and necrosis to similar levels. Moreover, further treatment of the Tdp1α-2a calli with NSC120686 did not cause any significant changes (*p* = 0.11) in the values of viable cells or PCD/necrotic events when compared to the untreated *Tdp1α*-depleted calli; though the values were statistically different when compared with the NT control. This could be because the viability of transgenic cells is already very low even without treatment, so it is difficult to perceive if the treatment had an effect in these conditions. Overall, these results indicate that the hTdp1 inhibitor NSC120686 may have an inhibitory effect on the plant Tdp1 activity.

### 3.2. Exposure of Medicago truncatula Calli to NSC120686 Results in Accumulation of DNA Damage

**Figure 2:**
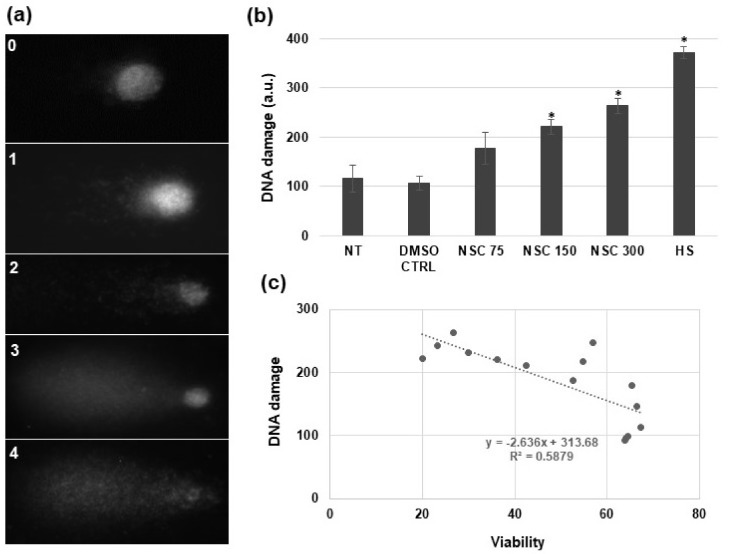
DNA damage induced by NSC120686 treatment in *M. truncatula* wild-type calli as assessed by alkaline single cell gel electrophoresis (SCGE) assay. (**a**) Nuclear morphology of the different comet classes; (**b**) Level of DNA damage (represented in a.u.) measured upon treatment with increasing NSC120686 concentrations (75 μM, 150 μM, and 300 μM). (**c**) Correlation analysis between the levels of DNA damage and cell viability. All data relative to DNA damage and cell viability (*n* = 36), including the controls, were used to build the correlation graphic. Asterisks (*) represent significant differences (*p* < 0.05) compared to NT, as evidenced by the *t*-Student test.

Following the finding that the highest NSC120686 concentration (300 μM) induced cell mortality at levels similar to those observed in the Tdp1α-depleted line, an SCGE analysis (or comet assay) was carried out to evaluate if this treatment also induces DNA damage. Under strong alkaline conditions (pH ≥ 13) the presence of both single strand breaks (SSBs), formed from alkali-labile sites, and double strand breaks (DSBs), is measured [27]. The results of the alkaline SCGE analysis carried out after four days exposure of *M. truncatula* calli to increasing concentrations of NSC120686 are showed in Figure 2. The results are presented as a.u., calculated following Formula (1), where the class number (c) ranges from 0 to 4 (Figure 2a). A significant accumulation of DNA damage was evident after treatment with 150 μM (221.2 ± 14.9 a.u.) and 300 μM (263.7 ± 16.2 a.u.) of NSC120686 (Figure 2b). Similar levels of DNA damage (alkaline SCGE, 198.2 ± 2.4 a.u.) were registered in the Tdp1α-depleted line, Tdp1α-2a in vitro grown plantlets [13]. In addition, the resulting values obtained from diffusion assay and comet assay were analyzed in order to establish what type of relationship might exist between the two sets of values. A negative correlation pattern (Figure 2c) emerged from this, as decreased viability was associated with enhanced accumulation of DNA damage.

### 3.3. In Silico Mining for Putative Interacting Partners of Tdp1

A bioinformatic analysis was carried out to identify putative interactors of the Tdp1 protein by using the STRING online tool (http://string-db.org/). This analysis was envisioned in order to assess the influence that the NSC120686 compound might have on putative Tdp1 networks in plants. As still little is known about these proteins in plants and the STRING database does not contain *M. truncatula* among its reference species, the analysis was performed using the human TDP1 protein sequence (#NP_001008744.1) as well as the Tdp1α protein sequence from *A. thaliana* (#OAO93376.1).

**Figure 3:**
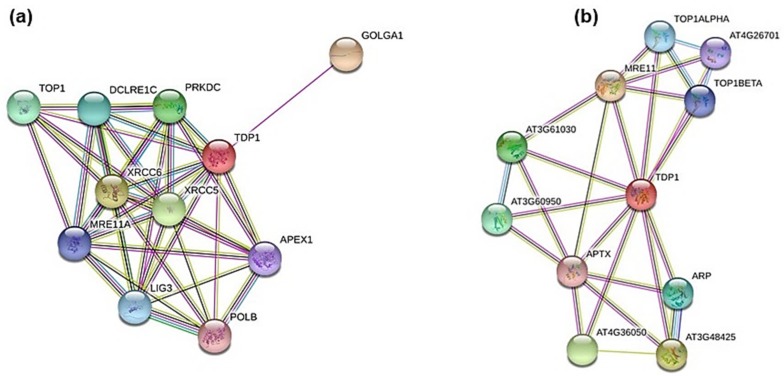
Putative protein-protein interactors of human (**a**) and *Arabidopsis thaliana* (**b**) Tyrosyl-DNA phosphodiesterase 1 (TDP1) as revealed by the STRING software analysis.

The STRING predicted interactors of hTdp1 are the following (Figure 3a):

- Golgin A1 (GOLGA1, #Q92805.3), probably involved in maintaining Golgi structure;
- X-ray repair complementing defective repair in Chinese hamster cells 5 and 6 (XRCC5, #P13010.3; XRCC6, #P12956.2), single-stranded DNA-dependent adenosine triphosphate (ATP)-dependent helicases involved in non-homologous end joining (NHEJ);
- Protein kinase, DNA-activated, catalytic polypeptide (PRKDC, #P78527.3), a serine/threonine-protein kinase that acts as a molecular sensor for DNA damage; involved in NHEJ;
- DNA Topoisomerase I (TOP1, #P11387.2), involved in the release of supercoiling and torsional tension of DNA introduced during the DNA replication and transcription by transiently cleaving and rejoining one strand of the DNA duplex;
- DNA cross-link repair 1C (DCLRE1C, #Q96SD1.2), involved in DSB repair mainly through NHEJ pathway;
- Ligase III, DNA, ATP-dependent (LIG3, #P49916.2), functions as a heterodimer with DNA-repair protein XRCC1 in the nucleus and can correct defective DNA strand-break repair and sister chromatid exchange following treatment with ionizing radiation and alkylating agents;
- Meiotic recombination 11 homolog A (MRE11A; #P49959.3), component of the MRN complex, plays a central role in DSB repair;
- APEX nuclease (multifunctional DNA repair enzyme) 1 (APEX1, #P27695.2), plays a central role in the cellular response to oxidative stress; functions as an apurinic/apyrimidinic (AP) endodeoxyribonuclease in the DNA base excision repair (BER) pathway;
- Polymerase (DNA directed) beta (POLB, #P06746.3), repair polymerase that plays a key role in BER pathway.

Similarly, the STRING predicted interactors of *Arabidopsis* Tdp1 are listed below (Figure 3b):

- MRE11 (#AT5G54260.1);
- DNAse I-like superfamily protein (#AT3G48425), apurinic/apyrimidinic (AP) endonuclease involved in active DNA demethylation and gene imprinting;
- Endonuclease 2 (#AT4G36050), exhibits apurinic/apyrimidinic (AP) endonuclease activity in vitro;
- Calcium-dependent lipid-binding (CaLB domain) family protein (#AT3G61030);
- C2 calcium/lipid-binding endonuclease/exonuclease/phosphatase (#AT3G60950);
- Apurinic endonuclease-redox protein (ARP, #AT2G41460.1), involved in the repair oxidative DNA damage and may act as a redox factor;
- DNA topoisomerase 1 alpha (TOP1ALPHA, #AED96613.1);
- DNA topoisomerase 1 beta (TOP1BETA, #AT5G55310.1);
- DNA binding (#AT4G26701), putative DNA topoisomerase type I;
- Aprataxin (APTX, #AT5G01310.1), DNA-binding protein involved in SSB repair, DSB repair, and BER.

Subsequently, using the human and *Arabidopsis* accessions, a BLAST search was carried out into the Phytozome database to identify *M. truncatula* accessions (Tables S1 and S2). The resulting accessions were used to examine the previously developed dataset of differentially expressed accessions in the Tdp1α-depleted line, Tdp1α-2a [13]. This led to identifying the following genes to be used as putative indicators of the *M. truncatula* response to NSC120686 treatment: *Top1β*, *Tdp2*, *Top2*, *XPF*, *MRE11*, *Rad50*, *Rad51-like*, *ERCC1*, *MUS81* (MUS81 Structure-Specific Endonuclease Subunit), *PARP1* (poly-(ADP-ribose) polymerase 1), *DRT100* (DNA-damage repair/toleration 100 protein), *DCLRP1A* (DNA cross-link repair protein 1A), and *ENDO3* (Endonuclease 3).

**Figure 4:**
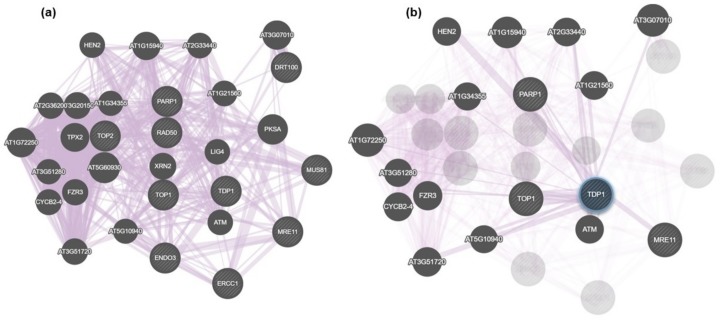
Putative interactive functional association network in *Arabidopsis thaliana*, as evidenced by GeneMANIA. (**a**) Overall connections at the level of the whole generated network. (**b**) Accessions co-expressed with Tdp1.

In addition to STRING, GeneMANIA was used to evidence putative interactive functional association network illustrating the relationships among genes. The analysis was mostly focused on co-expressed genes (Figure 4). While Figure 4a shows the connections at the level of the whole network generated in this search, Figure 4b focuses only on genes putatively co-expressed with *Tdp1*. These were the following: *PARP1, HEN2* (RNA helicase, ATP-dependent, SK12/DOB1 protein), *Top1*, *ATM* (Ataxia Telangiectasia Mutated serine/threonine-kinase), *MRE11*, *FZR3* (FIZZY-related 3), CYCB2-4 (Cyclin B2;4), AT3G07010 (pectin lyase-like superfamily), AT2G33440 (RNA-binding RRM/RBD/RNP family), AT1G15940 (Tudor/PWWP/MBT superfamily), AT1G34355 (Parallel Spindel 1 PS1 fork-associated (FDA) domain containing protein), AT2G21560 (nucleolar-like protein), AT1G72250 (di-glucose binding protein Kinesin motor domain-containing protein), AT3G51280 (tetratricopeptide (TPR)-like superfamily), AT5G51720 (WEB family containing the DUF827 domain), AT5G10940 (ASG2 transducin family protein, WD40 repeat family). Among them, *PARP1*, *HEN2*, *Top1*, *ATM*, *MRE11*, and AT1G15940 [28], have known roles in DNA repair. The resulting list contains additional accessions because the GeneMANIA software extends the user’s list to include genes that are functionally similar or that have shared properties with the initial query genes [26]. 

### 3.4. Expression Profiling of Medicago truncatula Tdp1 and Putative Interactors in Response to NSC120686 Treatment

**Figure 5:**
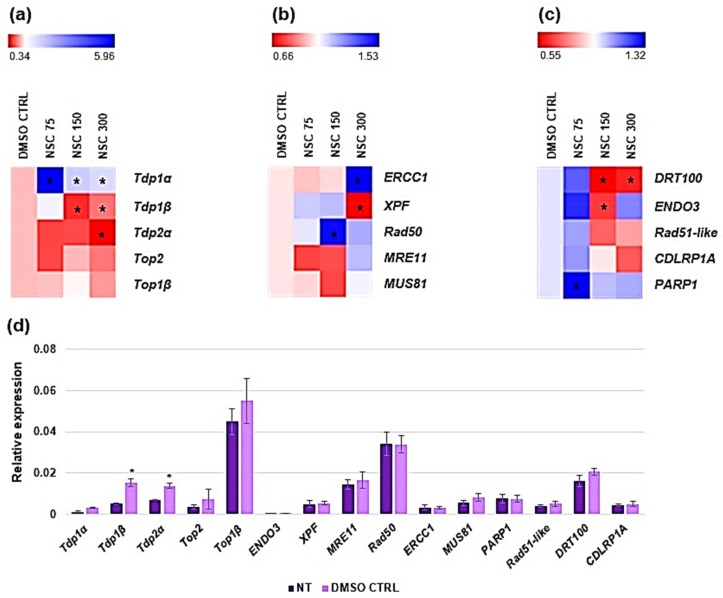
Expression profiles DNA damage repair (DDR) genes in response the increasing concentrations (75 μM, 150 μM and 300 μM) of NSC120686 delivered to *M. truncatula* calli for a four-day period. (**a**) Heatmaps grouping the *Tdp* and *Top* genes. (**b**) Heatmaps grouping genes belonging to alternative-Tdp1 repair pathways. (**c**) Heatmaps grouping potential Tdp1α interactors. (**d**) Expression profiles of DDR genes in NT and DMSO CTRL. Heatmaps were generated using MeV (Multiple Experiment Viewer) online software (http://mev.tm4.org). Data are presented as fold change to DMSO CTRL. Asterisks (*) indicates significant differences (*p* < 0.05) to control as revealed by the *t*-Student test.

As accumulation of DNA damage was observed in response to high concentrations (150 μM and 300 μM) of NSC120686, we investigated the expression profiles of genes with known functions in DNA damage repair (DDR). Because NSC120686 is an inhibitor of hTdp1 we evaluated the expression profiles of all *M. truncatula* Tdp genes and associated topoisomerases (Figure 5a), genes involved in putative alternative-Tdp1 repair pathways (Figure 5b), and genes associated with potential Tdp1 interactors (Figure 5c). Data are presented as heatmaps of values relative to DMSO CTRL whereas raw data are provided as Supplementary materials (Table S3). Generally, no differences were observed in gene expression levels when comparing the NT control with the DMSO CTRL, except for *Tdp1β* and *Tdp2α* (Figure 5d); in order to eliminate the putative influence of the DMSO, we decided to present the data as relative to DMSO CTRL.

The expression profiles of *Tdp1α* gene showed that this is significantly upregulated in response to all tested doses. Conversely, the *Tdp1β* and *Tdp2α* gene expression levels were significantly decreased at high NSC120686 concentrations (150 and 300 μM *Tdp1β*, and 300 μM for *Tdp2α*, respectively). On the other hand, no significant changes were observed in the case of *Top2* and *Top1β* gene expression (Figure 5a)*.*

When considering the expression profiles of alternative-Tdp1 repair pathways, a significant upregulation was observed in the case of *ERCC1* (at 300 μM of NSC120686) and *Rad50* (at 150 μM of NSC120686) genes, while *XPF* appears to be downregulated with the highest concentration (300 μM). No significant changes were evident in the case of *MUS81* and *MRE11* (Figure 5b).

Finally, the expression profile analysis of genes associated with potential Tdp1α interactors indicated significant downregulation of *DRT100* (at 150 and 300 μM NSC129686 concentrations) and *ENDO3* (at 150 μM NSC129686), and upregulation of *PARP1* gene at the lowest (75 μM) NSC120686 concentration. The expression levels of *Rad51-like* and *CDLRP1A* genes showed no significant changes compared to DMSO CTRL (Figure 5c).

**Figure 6:**
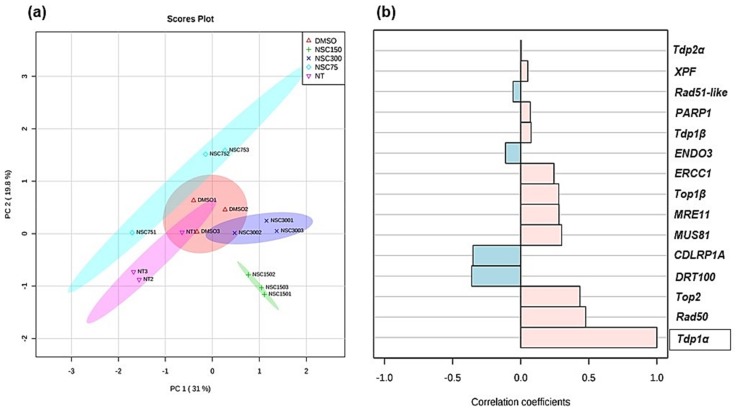
Correlation analysis of gene expression data in *M. truncatula* calli in presence/absence of NSC120686. (**a**) Principal component analysis 2D score plot. (**b**) Correlation pattern searching performed using the *Tdp1α* gene expression as the defining feature. Graphics were generated using the MetaboAnalyst (http://www.metaboanalyst.ca) online tool.

To ascertain the quality of the data, a PCA analysis was carried out using the three technical replicates of raw expression values (Table S3). This analysis evidenced a clear separation of the majority of the samples according to the given treatment (Figure 6a). In addition, a correlation pattern searching was carried out using the *Tdp1α* gene expression as the defining feature (Figure 6b). This was chosen mainly because *M. truncatula Tdp1α* gene is considered as the canonical isoform of this gene family as it shows higher similarity to the human *Tdp1* than the *Tdp1β* gene [10]. The results of the correlation pattern searching showed that most genes were positively correlated with the *Tdp1α* expression, while a negative correlation is evidenced in the case of *DRT100*, *CDLRP1A*, *ENDO3*, and *Rad51-like*, which belong to the category of putative interactors of Tdp1α (Figure 6b).

Overall, the expression profiling data show a complex picture of changes in *DDR* gene expression in response to NSC120686 treatment. Interestingly, while the *Tdp1α* gene is upregulated, the *Tdp1β* and *Tdp2α* are downregulated. Upregulation of *hTdp1* gene was observed also in human malignant cell lines subjected to NSC120686 treatment [8,9]. The fact that the two *Tdp1* genes present in *M. truncatula* have contrasting responses under exposure to NSC120686 further underlines the hypothesis that *Tdp1α* performs the canonical Tdp1 function, while *Tdp1β* might have additional/different functions [13,24].

## 4. Discussion

The NSC120686 compound was not long ago identified as an inhibitor of the hTdp1 enzyme [7]. It acts as a ligand that binds to the active site of the enzyme and thus, prevents its binding to other substrates. Since its identification, only a few studies have reported its use as a putative anticancer drug in combination with TopI-specific inhibitors [8,9]. This because, under this type of double treatment, the available Tdp1 enzyme responsible for the hydrolysis of the phosphodiesterase bond between TopI-enzyme and DNA is engaged with the false substrates whereas the DNA breaks produced by TopI inhibitors continue to accumulate and induce cell death. In addition, these studies also promote the *hTdp1* gene expression as a prognostic agent for malignant glioma because upregulation of the gene was associated with enhanced tumor aggressiveness [8,9,29] and the levels of *hTdp1* messenger RNA (mRNA) were inversely correlated with patient survival [29].

In plants, the Tdp1 functions are far less understood when compared to the advances in human Tdp1 research. The presence of a *Tdp1* gene family in plants (*Tdp1α*, *Tdp1β*) [10] adds another layer of complexity to this system and further expands the need to design dedicated studies to investigate their functions. Our previous studies have indicated an involvement of the *Tdp1* genes in DDR and stress response [10,12,13,14,18,23,24,30]. Furthermore, downregulation or mutations in the canonical *Tdp1α* gene resulted in the generation of dwarf plants in *M. truncatula* [13] and *Arabidopsis* [11], underlining its essential function in plant development.

In this study, the NSC120686 compound was used for the first time in plants to treat *M. truncatula* calli. At the protein level, plant and animal Tdp1 share the same domain organization and the catalytic sites along with the conserved amino acid residues (HKD-I and HKD-II) responsible for its function were evidenced also in plants [10,11]. Moreover, in *Arabidopsis*, it was proved that the recombinant Tdp1 protein was able to hydrolyze the 3′-phosphotyrosyl DNA substrates related to TopI-DNA induced damage [15]. Based on these premises, we hypothesized that the NSC120686, inhibitor of hTdp1 would have an effect also in plant cells. To evaluate what type of influence this compound have on plant cells, we measured several parameters, like cell viability, DNA damage, and expression of selective DDR genes. The data gathered from these analyses are discussed in view of the currently available literature from two other different systems, namely the in vitro grown Tdp1α-2a depleted line [13] and malignant human cell lines treated with NSC120686 [8,9].

We observed that the 300 μM NSC120686 concentration caused 50% of cell mortality in *M. truncatula* cells (Figure 1b). When considering the concentrations used to induce IC_50_ (50% cell death) in malignant cells lines, these ranged from 10 μM in IGROV-1 cell line [8] to 50 μM in U87 cell line [9]. Hence, a 6- to 30-fold increase in NSC120686 concentration was required to induce a cytotoxic effect in plant cells. Similarly, it was observed that camptothecin-treated carrot cell suspensions required up to 10-fold more compound to induce a damaging effect [31]. The same study showed that *tdp1β*-depleted carrot cells, presenting increased levels of PCD, were resistant to campthotecin (inhibitor of topoismonerase I) [31]. Also in our case, when the 300 μM NSC120686 was provided to the calli obtained from the Tdp1α-2a transgenic *M. truncatula* line, this did not cause any significant difference (*p* = 0.11) compared to the non-treated transgenic line; similarly, the PCD type of nuclei morphology are more prevalent. As the Tdp1α-2a lacks the *Tdp1α* function, this might indicate that the compound ‘attacks’ the Tdp1α in plants; on the other side, the Tdp1α-2a calli presented a low percentage of viable cell and enhanced PCD events even before treated with the compound, and these are comparable with the wild-type NSC120686-treated calli (300 μM) (Figure 1c). While necrosis is typically an acute cell death response that develops rapidly in response to stress, the PCD type of cell death may occur mainly during milder stresses [32,33]. On the other hand, it seems that the NSC120686-treated calli had a progressive and dose-dependent increase in the percentage of nuclei showing the necrosis type of morphology (Figure 1c). This is indicative of the fact that the treatment is perceived as a stressful situation.

In addition to cell mortality, the NSC120686-treated wild-type calli also showed enhanced levels of DNA damage (Figure 2b). Moreover, accumulation of strand breaks was also observed in the Tdp1α-2a transgenic line, characterized in a previous study [13]. Because DNA damage was evidenced in response to the NSC120686 treatment, we further evaluated the expression profiles of selective DDR genes (Figure 4), chosen based on a bioinformatic cross-search using online tools along with the previously generated RNA-seq dataset for the Tdp1α-2a [13]. A summary of these results, represented alongside with data from the currently available literature on plant and animal *Tdp1*, is provided in Table 2.

To start with, a very interesting result was the fact that the two *Tdp1* genes had a contrasting response, with *Tdp1α* being upregulated and *Tdp1β* being downregulated under NSC120686 treatment. This can be interpreted as a further indication that the *Tdp1α* and *Tdp1β* genes could have different functions. Other clues in this regard are the fact that: (1) in the Tdp1α-2a transgenic line, the observed up-regulation the *Tdp1β* gene was not able to compensate the lack of *Tdp1α* transcript [13], and (2) the *Tdp1β* gene seems to be more responsive at the early stages (first hours after treatment) of imposed stress, as evidenced in both *M. truncatula* and *A. thaliana* [24]. A subsequent observation would be that *Tdp1α* is the gene coding for the canonical Tdp1 protein; this observation is driven by the fact that also in human cell lines an upregulation of the *Tdp1* gene was evidenced in response to the NSC120686 treatment, concomitant with decreased protein levels [8,9] (Table 2). In *M. truncatula*, the observed upregulation of the *Tdp1α* gene could represent a negative feedback mechanism to compensate the functional impairment of the Tdp1 protein proposed as a possible effect of the NSC120686 treatment. On the other side, downregulation of the *Tdp2α* gene may be considered as an indicator for loss of cellular viability, as our previous results showed that overexpression of this gene was associated with an increased cell viability and proliferation [30]. Moreover, *Tdp2α* overexpression was correlated with enhanced DNA repair ability (mostly DSB repair, in agreement with its function in NHEJ pathway [34]) and tolerance to stress [18,23]. Conversely, the expression levels of DNA topoisomerases (*Tdp1β* and *Top2*) were not affected by the treatment with NSC120686, possibly suggesting that this compound does not induce topological changes in the DNA. 

In animal cells, Tdp1 is predicted to mainly function within the BER pathway [34,35] whereas other alternative-Tdp1 repair pathways could be involved the removal of Top1-mediated DNA damage as well [3,4]. Hence, we decided to investigate the expression levels of genes functioning in alternative pathways, such as *ERCC1* and *XPF*, playing essential roles in NER, or *MRE11*, *Rad50*, and *MUS81,* which act in DSB repair (HR and NHEJ). Our results showed that while *ERCC1* and *Rad50* genes were up-regulated, *XPF* gene was downregulated, and *MRE11* and *MUS81* reveal no significant changes in their expression levels under treatment with NSC120686. The situation was different when the expression profiles of these genes were searched in the Tdp1α-2a RNA-seq dataset [13] (Table 2). In this case, no significant changes in the transcript levels of *MRE11*, *Rad50*, *ERCC1*, *XPF*, and *MUS81* genes were observed, indicating that *M. truncatula* cells lacking the *Tdp1α* function do not rely on alternative repair pathways [13]. As the NSC120686 treatment induces increased expression of the *Tdp1α* gene, it is reasonable to find different expression of these genes when compared with a system lacking the *Tdp1α* function. Hence, our results suggest that treatment with NSC120686 influences not only the *Tdp1* genes but also other genes with roles in alternative repair pathways.

Similarly, when the profiles of putative Tdp1α interactors were investigated a divergent picture emerged while examining the NSC120686-treated calli and the in vitro grown Tdp1α-2a transgenic line (Table 2). The only gene that gave the same response under the two different conditions was *DRT100*, involved in the repair of abasic sites and SSBs during UV-induced DNA damage and associated with BER pathway [42]. An interesting point to discuss is the upregulation of both *Tdp1α* and *PARP1* at the lowest (75 μM) NSC120686 concentration when the levels of DNA damage and cell viability do not seem to be affected. Recent studies in animal cells suggested that PARP1 couples with Tdp1 for the repair of TopI–induced DNA damage [45]. A direct interaction between the N-terminal domain of Tdp1 and the C-terminal domain of PARP1 results in Tdp1 PARylation which leads to enhanced recruitment to DNA damage sites. Hence, in our situation, it is reasonable to hypothesize Tdp1 and PARP1 could be considered as interacting partners also in plant cells, and that *Tdp1α* and *PARP1* upregulation can prevent genotoxic damage at low concentrations of NSC120686. Differently, modified U87 glioma cells lines presenting either *Tdp1* overexpression or knockout did not reveal any significant changes in the expression patterns of *PARP1* gene [9]. Nonetheless, the case of Tdp1-PARP1 is far more complex as both are considered to be multipurpose proteins with roles covering DNA repair, chromatin remodeling, cell signaling and overall maintenance of genomic integrity [34,46].

In conclusion, the first study ever conducted in plants involving the use of the NSC120686 inhibitor of human Tdp1 activity shows that this compound is able to induce genotoxic damage and cell mortality when used at high concentrations. Even though the levels of cell death and accumulation of strand breaks are similar between the NSC120686-treated cells and the Tdp1α-2a transgenic line lacking the *Tdp1α* function, the molecular mechanisms behind these responses seem to be different. This is because, while the NSC120686 should affect the Tdp1α protein, the RNAi action on *Tdp1α* is performed at the mRNA level. The different mechanisms are evident as exemplified by the different patterns of expression of several genes with known functions in DNA damage response between the two systems. However, it cannot be ruled out that some of these divergences might be due to the use of different systems (calli vs. plants) or genomic variability; though, a recent study showed the occurrence of similar gene expression profiles in in vitro grown calli and *M. truncatula* plantlets [47]. Hence, the presented results are indicative of the use of hTdp1 inhibitor as a tool for future studies aiming to decipher the peculiar roles of the plant *Tdp1* genes.

## Figures and Tables

**Table 1:** Sequences of oligonucleotide primers used in the quantitative real-time polymerase chain reaction (qRT-PCR). Phytozome accession identifiers are provided.

Gene	Accession No.	Forward Primer (5′-3′)	Reverse Primer (5′-3′)
*Tdp1α*	Medtr7g050860	ACGAGTTGGGAGTGCTCTTT	GGGATTTATCCTTCGATTGTTT
*Tdp1β*	Medtr8g095490	GGTTGGTTTGAGCCATCTTT	GCAGGCACATTGTGATTTCT
*Tdp2α*	Medtr8g146980	CAGATGTTCAGCAAGGAACG	CCCGTCTTGCAAAGGATATT
*Top1β*	Medtr0172s0010	ATACACGTGGGCTATTGTCG	TCACTTGGATGAATGCGTT
*Top2*	Medtr3g103270	AGGATCCGTGGGATTGTAAGGC	ACAACAGAGAGGCCAGCCATAG
*Xpf*	Medtr5g013480	GGGTTCCGATGACGAAGTAT	CTCCACAGTCAAATCCTCCA
*MRE11*	Medtr2g081100	ATCCAAAGTGGTGCTGATGA	TGGATTCATTGTCCGAACTG
*Rad50*	Medtr3g084300.1	GGCGAGAAAGTTTGCCTTAG	GCCAATTTGCTTCATGTTGA
*ERCC1*	Medtr1g082570.1	CGTTCGTCAAATCCTCAGAA	TGAAGCTGCAGGAGCATTAT
*MUS81*	Medtr3g022850.1	AAGAAGCCACTGGATGTTCC	ATTTGGATGGCTTCTGGAAA
*Rad51-like*	Medtr4g124560.1	ATGGCTCATGCAACCACGAC	AACCTTGCTTCGGCTTCAGC
*PARP1*	Medtr1g088375	AAACCCACCCTCCTTCGTAGT	GTCCCTCGGTCTCTTTCCAA
*DRT100*	Medtr3g027940	ACCCTACCACGGCATCTTCA	TCTTCTGACTCGCCACGGAG
*DCLRP1A*	Medtr3g105470.1	TATGCGAGTCGGTTCAGCCT	AAGAAGGTGGCAGCAGGGTA
*ENDO3*	Medtr5g056160.1	CCTTGGTCGTCTGCTTTGCA	ATCGAGGAGCTGGTTGGTGT
*ELF1α*	Medtr6g021800	GACAAGCGTGTGATCGAGAGATT	TTTCACGCTCAGCCTTAAGCT

**Table 2:** Summary of expression profiles of selective genes with functions in DDR in three different systems: NSC120686-treated *M. truncatula* calli, Tdp1α-2a transgenic line with posttranscriptional downregulation of *Tdp1α* gene, and human malignant cell lines (IGROV-1 and U87) treated with NSC120686.

Gene	Function; DNA Repair Pathways	NSC120686	Tdp1α-2a [13]	IGROV-1/U87 [8,9]
*Tdp1α*	Repairs stalled TopI-DNA complexes; **BER** [34]	up-reg.	down-reg.	up-reg.
*Tdp1β*	Putatively repairs stalled topoisomerase I-DNA complexes; **putative BER** [10]	down-reg.	up-reg.	-
*Tdp2α*	Removal of DNA TopII-mediated DNA damage and cell signaling; **NHEJ** [34]	down-reg.	n.c.	n.d.
*Top1β*	Regulation of DNA topological state; cuts one of the two strands of double-stranded DNA [31]	n.c.	n.c.	n.d.
*Top2*	Regulation of DNA topological state; cuts both strands of the DNA helix simultaneously in order to manage DNA tangles and supercoils [36]	n.c.	down-reg.	n.d.
*Xpf*	Endonuclease that excises dimers; **NER** [37]	down-reg.	n.c.	n.d.
*MRE11*	Component of the MRN complex, central role in DSB repair; **HR**, **NHEJ** [38]	n.c.	n.c.	n.d.
*Rad50*	Component of the MRN complex, central role in DSB repair; **HR**, **NHEJ** [38]	up-reg.	n.c.	n.d.
*ERCC1*	Forms a catalytic complex with XPF; **NER** [37]	up-reg.	n.c.	n.d.
*MUS81*	Resolves recombination intermediates during DNA repair after inter-strand cross-links, replication fork collapse, and DNA double-strand breaks; **HR** [39]	n.c.	n.c.	n.d.
*Rad51-like*	DNA repair during meiosis; **HR** [40]	n.c.	down-reg.	n.d.
*PARP1*	Modifies various nuclear proteins by poly(ADP-ribosyl)ation, involved in the cell recovery from DNA damage; **BER** [41]	up-reg.	n.c.	n.d.
*DRT100*	Repair of abasic sites and SSBs during UV-induced DNA damage; **BER** [42]	down-reg.	down-reg.	n.d.
*DCLRP1A*	DNA interstrand cross-link repair and checkpoint-mediated cell cycle arrest [43]	n.c.	down-reg.	n.d.
*ENDO3*	T/G mismatch-specific endonuclease, nucleic acid binding, single-stranded DNA specific; **MMR** [44]	down-reg.	up-reg.	n.d.

Up-reg: Up-regulated; down-reg: Down-regulated; n.c.: No change in gene expression compared to respective control; n.d.: Not determined; gene expression not measured in the cited study; SSBs: Single strand breaks; MMR: Mismatch repair.

## Supplementary Materials

The following are available online at http://www.mdpi.com/2073-4425/9/4/186/s1, Table S1. Results of BLAST search performed in the Phytozome v12.1.5 database using the accessions of human Tdp1 putative interactors; Table S2. Results of BLAST search performed in the Phytozome v12.1.5 database using the accessions of *Arabidopsis* Tdp1α putative interactors; Table S3. Raw data of qRT-PCR relative expression used for PCA analysis.
